# Reliability of a Self-Administered Performance Evaluation Tool Based on the Modified Barthel Index

**DOI:** 10.7759/cureus.72665

**Published:** 2024-10-29

**Authors:** Hidehiro Watanabe, Yuka Oguri, Kento Noritake, Yohei Suzuki, Hidetoshi Okamura, Keisuke Fujii

**Affiliations:** 1 Department of Rehabilitation, Tokai Memorial Hospital, Kasugai, JPN; 2 Faculty of Health Sciences, Nihon Fukushi University, Handa, JPN; 3 Department of Rehabilitation Occupational Therapy Course, Faculty of Health Science, Suzuka University of Medical Science, Suzuka, JPN

**Keywords:** adl (activities of daily living), barthel index (bi), reproducibility of results, self-assessment tool, surveys and questionnaires

## Abstract

Background: Accurately assessing Activities of Daily Living (ADL) is crucial for evaluating patient independence, yet there is a lack of standardized tools suitable for large-scale surveys, particularly those that are self-administered. We aimed to validate the reliability of the self-administered Performance Evaluation Tool based on the modified Barthel Index (PET-MBI).

Methods: In this study, 53 patients and 18 occupational therapists were included to determine the inter-rater and intra-rater reliabilities via questionnaires. The inclusion criteria for patients were being admitted to a convalescent or community care ward and being able to communicate. The inclusion criteria for professionals were being an occupational therapist in charge of the patients and having at least two years of experience.

Results: The inter-rater reliability scores of PET-MBI were 80.4 ± 20.4 and 81.1 ± 19.2 points for patient and therapist ratings, respectively. The intraclass correlation coefficient (ICC) for the total score was 0.888. Regarding intra-rater reliability, the patients' scores were 80.4 ± 20.4 and 84.1 ± 19.8 points for the first and second assessments, respectively. The ICC for the total score was 0.927.

Conclusion: The self-administered PET-MBI demonstrated reliability in terms of total scores and numerous sub-items, demonstrating potential as a valuable tool for ADL assessment in large-scale surveys.

## Introduction

Activities of daily living (ADL) are closely linked to quality of life (QOL) [1-2] and death [3], and they serve as crucial indicators for evaluating the effectiveness of rehabilitation interventions and determining the need for care [4]. Proper ADL assessment enables a more accurate selection of rehabilitation programs and caregiving services, enhancing an individual's quality of life. Currently, various evaluation methods, such as the Functional Independence Measurement (FIM) [5] and the Barthel Index (BI) [6], which require external assessment, are commonly used for ADL assessment [7]. However, these methods require considerable human resources and time. Consequently, non-standardized evaluation methods are often employed in questionnaire surveys [8]. Although these methods assume independence in ADL in the target population and may not necessitate detailed assessments, a substantial number of older individuals do not fully maintain independence in ADL. Given the frequent use of questionnaire surveys by local governments and researchers, a standardized ADL assessment that is applicable even in survey settings would be advantageous. Therefore, there is a need to develop a standardized self-administered ADL assessment that allows individuals to evaluate their ADL abilities accurately. This need is particularly evident in our context, where we aim to conduct surveys on the post-discharge lives of patients to enhance in-hospital rehabilitation programs. However, the lack of a validated self-administered ADL assessment tool poses a significant challenge. Currently, no standardized self-administered ADL evaluation method exists that can be reliably used for such surveys. This gap in assessment tools limits our ability to efficiently gather comprehensive data on patients' functional status after discharge, which is crucial for improving rehabilitation strategies and ensuring better long-term outcomes.

The establishment of a self-administered ADL assessment method would enable individuals to assess their ADL abilities quantitatively and reduce the human resources and time required for assessments, allowing for more efficient evaluations. Moreover, it holds the potential for application in large-scale surveys. Self-reporting saves time and reduces cost, especially in large surveys and initial assessments; it also encourages rapid intervention and appropriate service provision. Therefore, the development and implementation of self-administered ADL assessments are expected to contribute to the establishment of more accurate and efficient assessment systems, ultimately improving the quality of services in the fields of caregiving and rehabilitation.

In this context, we focus on a performance evaluation tool based on the modified Barthel Index (PET-MBI) [9-11]. The PET-MBI is a Japanese-modified version of the BI developed in the United States, allowing quantitative assessment of 10 ADL items. The reliability and ease of capturing changes in ADL among stroke patients have been verified. The PET-MBI modified for a Japanese population has been confirmed to have reliability and validity [9-11]. If the reliability of the self-administered PET-MBI is verified, it can be utilized in large-scale surveys for ADL assessment, providing valuable information for caregiving services and rehabilitation. This study is the first attempt to validate a self-administered version of the PET-MBI, addressing a critical gap in ADL assessment methods. We hypothesize that the self-administered PET-MBI will demonstrate high reliability comparable to that of the version administered by healthcare professionals. The validation of a self-administered PET-MBI could revolutionize how we collect ADL data in large-scale studies and clinical practice, potentially leading to more personalized and effective support. Therefore, this study aimed to validate the reliability of the self-administered PET-MBI. Specifically, this study aimed to validate the reliability of the self-administered PET-MBI by assessing both inter-rater reliability between participants and professionals and intra-rater reliability across two self-assessments by participants.

## Materials and methods

Setting, design, and participants

This was a cross-sectional reliability study examining both inter-rater and intra-rater reliability using intraclass correlation coefficients (ICC) and weighted kappa coefficients. The participants in this study were patients who were hospitalized at a facility affiliated with the first author from April to June 2023 and their respective occupational therapists. The hospital to which the first author is affiliated is a medium-sized institution with 190 beds located in Japan. The facility has 50 beds in the recovery rehabilitation ward, 95 beds in the community comprehensive care ward, and 45 beds in the general ward.

The inclusion criteria for patient participants were as follows: The participants were defined as those who were admitted to a recovery rehabilitation ward or a community comprehensive care ward during the study period and those who were able to communicate with others. Exclusion criteria comprised individuals with a Mini-Mental State Examination (MMSE) [12] score below 18, those facing challenges in MMSE implementation, individuals with writing difficulties, and those for whom the investigation posed challenges due to a short hospitalization period. We excluded participants with an MMSE score below 18 points, following the criteria used in the study that assessed the reliability and validity of the PET-MBI [10]. Inclusion criteria for professionals were that they were the occupational therapist in charge of the patient and that they had at least two years of experience. Consequently, the selection of occupational therapists was not randomized. Before the study began, a training session on the PET-MBI was held to ensure that occupational therapists could score uniformly.

Participants conducted two self-assessments of their ADL using the PET-MBI, with an interval of 3-4 days. Professionals performed their assessments on the same day as the participants' initial evaluations. To ensure unbiased evaluations, PET-MBI results from both the participants and their occupational therapists were blinded. After explaining the research and obtaining consent, patients were given a minimal explanation: 'This is a form to score conditions related to daily living activities, such as eating and toileting. Please follow the instructions on the evaluation form and answer the questions.

Procedure

The PET-MBI is recognized as a robust method for assessing ADL. This represents a modification of the Japanese version of the MBI developed by Shah et al. [13], and its validity and reliability confirmed by Ohura et al. [9-11]. The PET-MBI includes the same categories as the BI, including personal hygiene, self-bathing, feeding, using the toilet, stair climbing, getting dressed, bowel control, bladder control, chair/bed transfer, and ambulation. However, it incorporates Japanese habits (such as the use of chopsticks and bathing in a tub), and the scoring for each item differs slightly from that of the BI. For instance, in the BI, eating was scored as 10, 5, or 0 points, whereas in the PET-MBI, it was scored as 10, 8, 5, 2, or 0 points, allowing for a more detailed assessment of the level of assistance. The scoring range is 0 to 100, with higher scores indicating greater independence in ADL. The occupational therapist scored the PET-MBI based on the participant's daily activities. The patients scored the PET-MBI on their own.

Demographic information of the participants, age, sex, diagnosis, and MMSE scores were obtained from medical records. Each assigned occupational therapist's years of experience were also recorded.

Data analysis

This study investigated inter-rater and intra-rater reliabilities using IBM Corp. Released 2021. IBM SPSS Statistics for Windows, Version 28.0. Armonk, NY: IBM Corp. for all statistical analyses.

To assess the inter-rater and intra-rater reliabilities of the PET-MBI, intraclass correlation coefficients (ICC) were calculated for total scores. Additionally, weighted kappa coefficients were calculated for each sub-item score. The statistical significance of the weighted kappa coefficient was determined using a chi-square test.

Ethical considerations

This study was approved by the hospital’s Research Ethics Committee, to which the first author is affiliated (2022-004). The purpose and methods of the study were conveyed to the participants and their respective occupational therapists through both written and oral means, and written informed consent was obtained before the commencement of the study.

## Results

A total of 53 patients (mean age 78.6 ± 11.6 years, 32 females) were included in this study. The patients had diagnoses of cerebrovascular disease (n = 14), musculoskeletal disorders (n = 32), cardiovascular disease (n = 1), respiratory disease (n = 1), and disuse syndrome (n = 5) (Table 1). The average MMSE score was 26.3 ± 3.2. Moreover, 18 occupational therapists conducted the assessments (mean experience of 6.9 ± 4.4 years).

**Table 1:** Background characteristics of the patients (N=53) SD: Standard deviation, MMSE: Mini-mental state examination, MMSE scores range from 0 to 30, with higher scores indicating better

Item	n (%)
Age, years, mean ± SD	78.6 ± 11.6
Female	32(60.4)
Medical disease	
Cerebrovascular disease	14(26.4)
Locomotor disease	32(60.4)
Cardiovascular disease	1(1.9)
Respiratory disease	1(1.9)
Disuse syndrome	5(9.4)
MMSE, score, mean ± SD	26.3 ± 3.2

Inter-rater reliability

The total scores for PET-MBI were 80.4 ± 20.4 points for patient ratings and 81.1 ± 19.2 points for occupational therapist ratings (Table 2). The ICC for the total score was 0.888. The weighted kappa coefficients for each sub-item ranged from 0.344 (feeding) to 0.676 (chair/bed transfer) (p < 0.001) (Table 3).

**Table 2:** Patients' and occupational therapists' PET-MBI assessment results SD: standard deviation, PET-MBI, Performance Evaluation Tool based on the modified Barthel Index, PET-MBI total score ranges from 0-100, with a higher score indicating better. Score range for each item: Personal hygiene (0-5), Self-bathing (0-5), Feeding(0-10), Using the toilet(0-10), Stair climbing(0-10), Getting dressed(0-10), Bowel control(0-10), Bladder control(0-10), Chair/Bed transfer(0-15), Ambulation(0-15). Patients' PET-MBIs were interval 3-4 days between the first and second assessments.

Item	Occupational Therapist	Patient	
		First assessment	Second assessment
	Mean ± SD	Mean ± SD	Mean ± SD
PET-MBI total score	81.1 ± 19.2	80.4 ± 20.4	84.2 ± 19.8
Personal hygiene	4.6 ± 0.7	4.3 ± 1.2	4.6 ± 0.8
Self-bathing	3.3 ± 1.3	3.4 ± 1.8	3.7 ± 1.2
Feeding	9.6 ± 1.0	9.0 ± 2.3	9.2 ± 1.7
Using the toilet	8.5 ± 2.4	8.4 ± 2.4	8.6 ± 2.6
Stair climbing	4.4 ± 3.7	4.8 ± 4.3	5.9 ± 4.2
Getting dressed	8.5 ± 2.4	8.3 ± 2.7	8.9 ± 2.4
Bowel control	8.7 ± 2.8	8.3 ± 2.8	8.7 ± 2.3
Bladder control	8.4 ± 3.0	8.7 ± 2.6	8.8 ± 2.4
Chair/Bed transfer	13.2 ± 3.1	13.0 ± 3.7	13.3 ± 2.9
Ambulation	11.9 ± 4.3	11.9 ± 4.3	12.2 ± 4.6

**Table 3:** Inter-rater reliability and intra-rater reliability ICC: Intraclass correlation coefficient, CI: Confidence interval, OT: Occupational therapist, the statistical significance of the weighted kappa coefficient was determined using a chi-square test.

Item	Inter-rater reliability		Intra-rater reliability	
	Patient's 1st assessment - OT assessment	p-value	Patient's 1st assessment - Patient's 2nd assessment	p-value
ICC (95%CI)	0.888 (0.814–0.934)		0.926 (0.875–0.956)	
Cronbach α	0.940		0.971	
Weighted kappa				
Personal hygiene	0.489	< 0.001	0.400	< 0.001
Self-bathing	0.528	< 0.001	0.535	< 0.001
Feeding	0.344	< 0.001	0.480	< 0.001
Using the toilet	0.604	< 0.001	0.733	< 0.001
Stair climbing	0.563	< 0.001	0.635	< 0.001
Getting dressed	0.656	< 0.001	0.698	< 0.001
Bowel control	0.575	< 0.001	0.694	< 0.001
Bladder control	0.512	< 0.001	0.535	< 0.001
Chair/Bed transfer	0.676	< 0.001	0.673	< 0.001
Ambulation	0.512	< 0.001	0.666	< 0.001

Intra-rater reliability

The total scores for PET-MBI were 80.4 ± 20.4 points for the first assessment and 84.1 ± 19.8 points for the second assessment by patients (Table 2). The ICC (2, 1) of the total score was 0.927. The weighted kappa coefficients for each sub-item ranged from 0.400 (personal hygiene) to 0.733 (using the toilet) (p < 0.001) (Table 3).

## Discussion

In this study, we investigated the reliability of the self-administered PET-MBI. The results confirmed reliability both within and between raters.

The inter-rater reliability of the total score on the PET-MBI showed an ICC of 0.888, whereas the intra-rater reliability demonstrated an ICC of 0.927. Both values exceeded 0.810, indicating excellent reliability [14]. A previous study reported an ICC of 0.651 for the total score of the patient's self-administered FIM and the total score scored by the expert [15]. Although the scale used in the present study was different from that in the aforementioned study, the ICC of the total score was higher. Furthermore, the ICC for inter-rater reliability in the present study was 0.888, which surpasses the previous study. This indicates that the PET-MBI used in this study may be more reliable than the FIM, possibly due to the fact that the PET-MBI has 10 fewer items than the FIM because the BI is modified.

The weighted kappa coefficients for inter-rater reliability in the PET-MBI sub-items ranged from 0.34 for feeding (fair) to above 0.41 (moderate) for others. Occupational therapists assigned an average score of 9.6 ± 1.0 points for feeding. In comparison, patients were assigned an average score of 9.0 ± 2.3 points, indicating lower scores given by patients compared to those assigned by occupational therapists. In our hospital, meal service methods are not standardized, and staff members assist with tasks such as removing lids or opening sauce packets, irrespective of the patient's ability. It is possible that patients recognized assistance and rated themselves lower. In addition, a previous study examining the inter-rater reliability of a self-administered FIM also found a low ICC of 0.12 for the sub-item of feeding [15]. In occupational therapists, feeding is an ADL ability that they often assist with. In the future, it may be necessary to develop a highly reliable assessment of feeding ability by self-administration.

The weighted kappa coefficient for intra-rater reliability was 0.40 (fair) only for personal hygiene and above 0.41 (moderate) for other sub-items. Regarding personal hygiene, patients were assigned average scores of 4.3 ± 1.2 points for the first assessment and 4.6 ± 0.8 points for the second assessment. The personal hygiene category has four points with the expression "can maintain personal hygiene independently but requires minimal assistance or supervision before and after implementation," without specifying the content of minimal assistance. Therefore, confusion during patient scoring and potential changes in scores were considered. No studies were found that examined the intra-rater reliability of patients' own self-administered assessments of ADLs. Therefore, it is necessary to be cautious when interpreting the results of personal hygiene. Conversely, the weighted kappa coefficients were 0.41 or higher, except for personal hygiene, suggesting that the PET-MBI could be evaluated by the patients themselves if problems with personal hygiene were solved. To validate these points, further study is needed.

This study has five major limitations. First, it was conducted at a single institution. Although the participating hospital is a typical convalescent rehabilitation hospital, patient characteristics and therapists' experiences may vary across different facilities. Therefore, further research is needed to validate the findings in multiple institutions. Second, the study population was limited to patients with an MMSE score of 18 or higher. This criterion was implemented based on previous research to ensure the validity of self-reported surveys [10]. Consequently, a significant limitation of this study is that its findings cannot be applied to patients with MMSE scores below 18. Third, this study did not estimate the sample size. The validity of the sample size comprising 53 patients and 18 occupational therapists who met the inclusion and exclusion criteria within the study period cannot be guaranteed, which is a major issue. However, we believe that the extremely high ICC values indicate the feasibility of self-administered PET-MBI. Fourth, this study was conducted on hospitalized patients. Our goal is for the PET-MBI to eventually be performed in the community by people who need care in a self-administered form. Therefore, future studies should be conducted on community-residing patients who require nursing care. We will need to collaborate with other facilities to verify the results of this study. Fifth, the patients in this study were a group with relatively high ADL (Activities of Daily Living) scores. This suggests that caution is necessary when applying this approach to patients with moderate or severe ADL impairments. These limitations highlight the need for caution when generalizing the results and underscore the importance of conducting broader, multi-center studies in the future to enhance the applicability of the findings.

## Conclusions

The study has explored the reliability of the self-administered PET-MBI, indicating a generally high reliability in total scores and many sub-items, akin to those administered by healthcare professionals. These findings partially support our hypothesis and suggest that the self-administered PET-MBI could enhance ADL data collection in large-scale studies and clinical practice, potentially leading to more personalized and effective support. However, observed variabilities in inter-rater reliability for feeding and intra-rater reliability for personal hygiene underline the need for careful application. Future applications might benefit from focusing on the total score or selectively using specific items to optimize utility. Such adjustments could help improve the assessment process, aiming for efficiency and adaptability in various clinical and research environments.
